# Transplanted artificial amnion membrane enhanced wound healing in third-degree burn injury diabetic mouse model

**DOI:** 10.1016/j.reth.2024.03.016

**Published:** 2024-03-26

**Authors:** Kenichi Arai, Satoshi Yoshida, Etsuko Furuichi, Shintaroh Iwanaga, Tanveer Ahmad Mir, Toshiko Yoshida

**Affiliations:** a Department of Clinical Biomaterial Applied Science, Faculty of Medicine, University of Toyama, Toyama, Japan; b Department of Biotechnology, Faculty of Bioresource Sciences, Akita Prefectural University, Akita, Japan; c Department of Medical Oncology, Toyama University Hospital, Toyama, Japan; d Division of Biomedical System Engineering, Graduate School of Science and Engineering, University of Toyama, Toyama, Japan; e Tissue/Organ Bioengineering and BioMEMS Lab, Organ Transplant Centre of Excellence (TR&I Dpt), King Faisal Specialist Hospital and Research Centre, Riyadh 11211, Saudi Arabia

**Keywords:** Artificial amniotic membrane, Wound healing, Burn injuries, Diabetes, Ulcers, Wound dressing

## Abstract

**Introduction:**

Wound healing is severely compromised in patients with diabetes owing to factors such poor blood circulation, delayed immune response, elevated blood sugar levels, and neuropathy. Although the development of new wound healing products and prevention of serious complications such as infections in wounds have received substantial interest, wound healing remains a challenge in regenerative medicine. Burn wounds, especially third-degree burns, are difficult to treat because they are associated with immune and inflammatory reactions and distributive shock. Wound care and treatment that protects the burn site from infection and allows wound healing can be achieved with bioengineered wound dressings. However, few studies have reported effective dressings for third-degree burn wounds, making it important to develop new dressing materials.

**Methods:**

In this study, we developed an artificial amniotic membrane (AM) using epithelial and mesenchymal cells derived from human amnion as a novel dressing material. The artificial AM was applied to the wound of a diabetic third-degree burn model and its wound healing ability was evaluated.

**Results:**

This artificial amnion produced multiple growth factors associated with angiogenesis, fibroblast proliferation, and anti-inflammation. In addition, angiogenesis and granulation tissue formation were promoted in the artificial AM-treated mouse group compared with the control group. Furthermore, the inflammatory phase was prolonged in the control group.

**Conclusions:**

Our preliminary results indicate that the artificial AM might be useful as a new dressing for refractory ulcers and third-degree burns. This artificial AM-based material represents great potential for downstream clinical research and treatment of diabetes patients with third-degree burns.

## Highlights

• An artificial amniotic membrane (AM) was developed as a new wound dressing material.
• The AM released angiogenesis, fibroblast growth, and anti-inflammation factors.
• The AM suppressed inflammation and promoted wound healing in diabetic mice.
• Wound closure was faster and granulosa tissue was thicker in AM-treated mice.
• This new AM has great potential for downstream clinical research on burn injuries.

## 1 Introduction

Third-degree burns are severe and involve the complete destruction of all layers of the skin. In patients with diabetes, third-degree burns not only obstruct their blood circulation but also affect cell viability in the tissues. Therefore, the wound healing process of a burn wound may be delayed in diabetes patients compared with healthy individuals [1]. Additionally, wounds in diabetes patients have a high risk of infection and can become chronic [2,3]. Infected or necrotic wounds require removal during treatment. For example, 7–20% of patients with diabetic foot ulcers may require surgical excision (amputation) of a toe, foot, or part of a leg. In worst-case scenarios, diabetic foot ulcer can become life-threatening [4,5], as it results in impaired blood flow (obstruction of the supply of nutrients and oxygen), reduced ability to synthesize proteins due to persistent high blood sugar, and weakened immune cell function [6,7]. The inflammatory response at the wound site is often delayed because the migration of neutrophils and macrophages to the wound site is prolonged in diabetes patients, which in turn slows down re-epithelialization and the transition of the inflammatory phase to the anti-inflammatory and proliferative phases [8]. Furthermore, patients with diabetes often suffer from peripheral neuropathy, which delay the recognition of injury caused by external factors, further exacerbating the wound.

For patients with underlying diabetes mellitus suffering from burns and other severe wounds, it is important to use proper dressing material that prevents infection and promotes wound healing. The use of new dressing materials that contain mesenchymal cells has been reported to promote wound healing in the treatment of chronic wounds such as diabetic foot ulcers [9,10]. However, all existing dressing materials have low efficacy and several drawbacks. Therefore, new materials with sufficient anti-inflammatory and antimicrobial properties are urgently needed.

Amniotic membrane could be investigated as an alternative dressing material because of its anti-inflammatory and antimicrobial properties. Moreover, mesenchymal and epithelial cells derived from amniotic membranes can differentiate into various tissues, contributing to wound healing. Further, amniotic membrane is less likely to be rejected as a graft material in both autologous and allogeneic transplantations [11]. Although it is still not entirely understood how amniotic tissue factors influence the wound healing process at the molecular level, amniotic membranes contain several growth factors such as PDGF, bFGF, and EGF, which promote angiogenesis, fibroblast proliferation, and epithelialization during the wound healing process [[12], [13], [14]]. In addition, amnion-derived epithelial cells produce antimicrobial molecules such as β-defensin-3, which may reduce the risk of infection in wound patients [15]. Because of these inherent characteristics, amnion membrane can be useful as a wound dressing material for third-degree burn injuries in patients with diabetes.

A major challenge while developing amnion-based regenerative biomaterials is that the amount of amnion-derived mesenchymal cells in the connective tissue of amniotic membranes is often low [16]. Additionally, fresh human amnion is difficult to obtain. Other problems associated with amniotic membranes include low viability of amnion-derived cells and low mechanical strength of lyophilized amnion [17]. Recently, cryopreserved amnion has been used as an ocular surface graft for patients with ocular disease, but the survival rate of amnion-derived cells is very low [18]. To address these issues, we previously developed ultra-dry human amniotic membrane (HD-AM), which retains several bioactive substances such as cytokines [19]. Although HD-AM can be used as a dressing material in emergency clinical situations and has several advantages as a medical material, the activity of physiologically active substances in HD-AM is limited and the cells in HD-AM are not viable.

Thus, in the present study, we developed an artificial amniotic membrane (AM) by incorporating the vitrigel with the immortalized human amniotic epithelial cells (ihAEs) and the immortalized human amniotic mesenchymal cells (ihAMs) as a new dressing material. The efficacy in wound healing of artificial-AM was evaluated *in vitro* experiments. Additionally, we examined the wound healing effect of the newly constructed material on a diabetic mouse model with third-degree burn. The proposed biomedical material showed functional effects both *in vitro* and *in vivo*, implying that artificial amniotic membrane might be an ideal tool for wound healing studies and clinical translation.

## 2 Materials and methods

### 2.1 Cell preparation

Immortalized human amniotic epithelial cells (ihAEs) and immortalized human mesenchymal cells (ihAMs) were established from original amniotic cells according to previously described methods [20,21]. Briefly, hAEs and hAMs were stably transfected with human papillomavirus type16E6 and E7 (HPV16E6/E7) and human telomerase reverse transcriptase (hTERT) genes by retrovirus infection. The ihAEs and the ihAMs have better proliferative properties than it of primary cells. In addition, these cells have the stem cell properties and the production capacity of anti-inflammatory related humoral factors as well as primary cells [22]. ihAEs were cultured in a Dulbecco's Modified Eagle's Medium (DMEM)/Nutrient Mixture F-12 Ham medium (Sigma-Aldrich, St. Louis, MO, USA) supplemented with 10% heat-inactivated fetal bovine serum (FBS) and antibiotic solution (100 U/mL penicillin and 0.1 mg/mL streptomycin; No. 2625384, Nacalai Tesque, Kyoto, Japan). ihAMs were cultured in DMEM (Sigma-Aldrich) supplemented with 10% FBS, antibiotic solution, and 2 mM l-glutamine solution (Nacalai Tesque). Adult human dermal fibroblasts (HDFa) were purchased from Thermo Fisher Scientific (Waltham, MA, USA) and cultured in Human Fibroblast Expansion Medium (Medium 106; Thermo Fisher Scientific). Human umbilical vein endothelial cells (HUVECs) were purchased from Lonza, Inc. (Walkersville, MD, USA) and were cultured in endothlial cell growth medium-2 (EBM-2; Lonza, Inc.). All cell types were cultured under a humidified 37 °C and 5% CO_2_ condition.

### 2.2 Fabrication of artificial amniotic membrane (AM) and preparation of artificial-AM culture medium

**Fig. 1:**
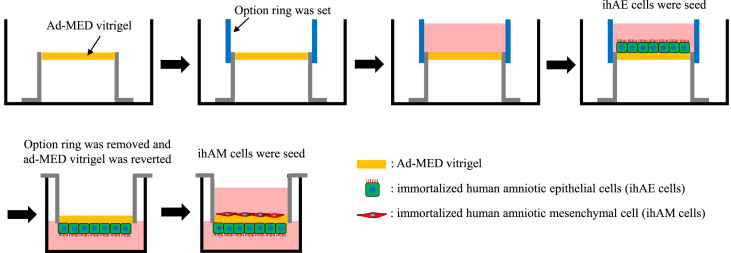
The procedure for developing the artificial amnion membrane. First, the ad-MED vitrigel was placed in a culture dish, followed by seeding immortalized human amniotic epithelial cells (ihAEs) after setting an option ring on the ad-MED vitrigel. Following the adhesion of ihAEs to the vitrigel, the option ring was removed and the vitrigel was inverted. Immortalized human amniotic epithelial cells (ihAMs) were then seeded on the opposite side of the vitrigel. The cells were continued to be cultured for further observations.

A collagen xerogel membrane chamber (ad-MED Vitrigel™) and option ring were purchased from Kanto Chemical (Tokyo, Japan). The option ring set around ad-MED Vitrigel (Fig. 1) [23]. A 0.5 mL cell suspension solution (100,000 cells/mL) of ihAMs was seeded and cultured on the option ring side for 24 h at 37 °C. The ihAM cells were labeled with the fluorescent vital red dye (CellBrite Red Cytoplasmic Membrane Dye, Biotium Inc., Fremont CA, USA) according to the manufacturer's instructions. After cultivation, the option ring was removed from ad-MED Vitrigel. Subsequently, the ad-MED Vitrigel was reversed and transferred to a 12 well-plate. A 0.5 mL cell suspension solution (200,000 cells/mL) of ihAEs was seeded and cultured for 24 h at 37 °C. The ihAM cells were labeled with a fluorescent vital green dye (CellBrite Green Cytoplasmic Membrane Dye). To evaluate the culture supernatant of artificial AM, the artificial AM was cultured in 0% FBS/DMEM for 48 h.

### 2.3 Air-liquid interface culture

The air-liquid interface system of ad-MED Vitrigel has been previously reported [24,25]. The ihAE cells suspended in 0.5 mL of the medium at 200,000 cells/mL were seeded and cultured onto the ad-MED Vitrigel of a chamber preset in a 12 well-plate with each well containing 1.5 mL of the medium. After 24 h, the exterior and interior media in the chamber were changed to CnT-Prime Epithelial 3D Airlift Medium (CELLnTEC, Bern, Switzerland), and ihAEs were cultured for 24 h. After cultivation, the interior medium in the chamber was removed to start the additional culture under the air-liquid interface for 21 days. The exterior medium was replaced every 3 days.

### 2.4 Protein array

The proteins of the artificial AM were extracted with RIPA buffer (Nacalai Tesque, Japan) and a 1% protease inhibiter cocktail (Nacalai Tesque). The protein quantification was measured by using protein assay BCA kit (Nacalai Tesque). To confirm the expression of proteins in the extracted artificial AM, a protein array was performed with RayBio *C*-Series Human Cytokine Antibody Array C5 kit (RayBiotech, Norcross, GA) according to the manufacturer's instructions. The membranes were visualized using an LAS-4000 luminescent image analyzer (Image Quant LAS4000, Fujifilm, Japan) for detection. The densities of protein expression plots were measured using the Image J software and analyzed using the AAH-CYH-5 analyze software.

### 2.5 Endothelial cell migration assay

The migration of HUVECs (Lonza, Inc. Walkersville, MD, USA) was evaluated *in vitro* using a scratch wound healing assay. Briefly, HUVECs were seeded and cultured at 50,000 cells/cm^2^ on 24 well-plates for 24 h. After cultivation, a liner wound was drawn vertically to a horizontal line using a 200 μL pipette tip and a ruler. The sample was washed 3 times with serum-free medium (DMEM), and 10% FBS/DMEM, artificial-AM culture medium, or 0% FBS/DMEM was added to each well. The wound area filling was observed, and images were taken under an inverted phase contrast microscope (Keyence, Osaka, Japan) at 0, 3, 6, 9, 12, 15, 18, 21, and 24 h after the scratch. Wound area filling by migrating cells was analyzed using Image J [26].

### 2.6 *In vitro* proliferation of fibroblasts

HDFa (Thermo Fisher Scientific) were seeded and cultured at 15,625 cells/cm^2^ on 96 well-plates for 24 h. After cultivation, the sample was washed 3 times with serum-free medium (DMEM), and 10% FBS/DMEM, artificial-AM culture medium, or 0% FBS/DMEM was added to each well. After 72 h of cultivation, CyQuant assay (Molecular Probes CyQuant; Invitrogen, Carlsbad, CA, USA) was performed to quantify the number of cells.

### 2.7 Establishment of a third-degree burn injury model of diabetic mice

We used 8-week-old male C57BLKS/J Iar- + Leprdb/+Leprdb (db/db) mice, which is a diabetic mouse model (Japan SLC, Inc), for the animal experiments. All experiments were performed in accordance with the recommendations of the Guide for the Care and Use of Laboratory Animals (National Institutes of Health). All experimental protocols were approved by the University of Toyama Experiment Facility (A2022MED-24). A third-degree burn injury model was established following our previously reported methods [27]. Briefly, mice were anesthetized by intraperitoneal injection of an anesthetic (a mixture of 4 mg/kg midazolam, 3 mg/kg medetomidine chloride, and 5 mg/kg butorphanol tartrate sodium pentobarbital). Their dorsal hairs were clipped and depilated using a hair removal cream. Then, a circular area (diameter, 10 mm) on the dorsal skin was exposed to hot water (90 °C) for 10 s in a 1.5 mL reaction tube, which was cut at the bottom. The entire skin layer at this site was excised and used as an open wound to examine the healing process.

### 2.8 Application of artificial AM to the third-degree burn injury diabetic mice model

A circular artificial AM (diameter, 10 mm) was placed on the skin excision site (artificial-AM group, n = 6). In the control group (n = 6), artificial AM was not used. After treatment, the skin in both groups was covered with a food plastic wrap (Tegaderm™ Diamond transparent film; 3 M, St.Paul, MN, USA) and a stainless mesh (0.06 mm, 150 ms^−1^; Kyuuhoukinzoku, Osaka, Japan). A total of six mice from each group were prepared for subsequent analyses on post-operative day (POD) 5, 9, and 14. We previously reported that a sample size of 6 mice per group is optimal for wound healing investigations [27]. Therefore, in the present study, we used a sample size of 6 mice to examine the wound healing efficacy of artificial AM on third-degree burn in diabetic animal model.

### 2.9 Histological and immunohistochemical analysis

The artificial AMs were fixed in 4% paraformaldehyde in a phosphate-buffered saline (PBS) solution for 24 h. The fixed samples were then embedded in a 7 wt% agar solution (Difco Agar Noble; Becton Dickinson, Franklin Lakes, NJ, USA). Subsequently, the samples were embedded in an optimal cutting temperature compound (SCEM; Section-Lab Co. Ltd., Hiroshima, Japan) and cryopreserved. The embedded samples were sliced into 20-μm thick cryosections using a cryostat (Leica CM3050S; Leica Microsystem K.K, Tokyo, Japan).

**Fig. 2:**
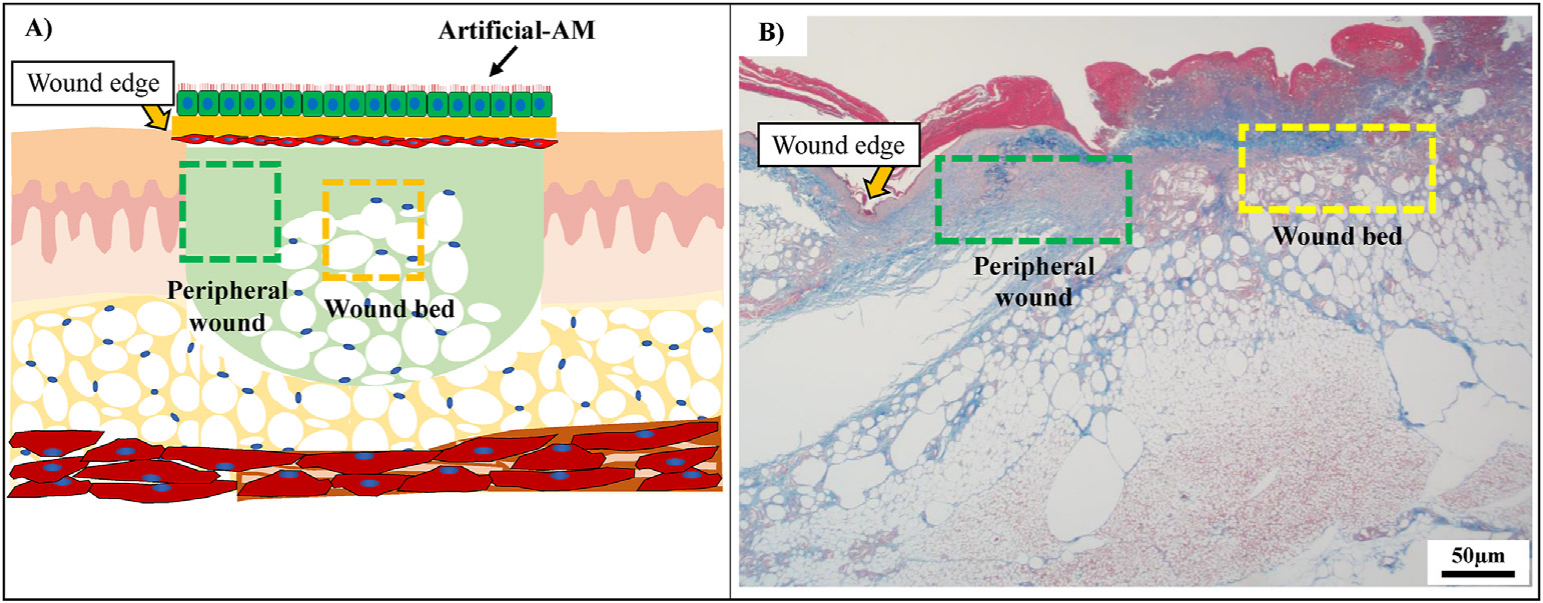
The observation area in granulation tissue area in the wound site. (A) Conceptual illustration of the wound site in third-degree burn injury diabetic mouse model. (B) Histological image of the third-degree burn injury (B). Scale bars, 50 μm.

The artificial AMs and the collected tissues were fixed in 4% paraformaldehyde in a PBS solution for 48 h. The samples were then embedded in paraffin and cut into 4-μm sections. The sections were used for hematoxylin and eosin (HE) staining, azan staining, orcein staining, and immunostaining. CK14 (dilution 1:100), CD59 (dilution 1:10,000),α-SMA (dilution 1:2500), CD31 (dilution 1:4000), Iba-1 (dilution 1:2000), and CD163 (dilution 1:500), purchased from Abcam (Cambridge, UK), were used as the primary antibodies. Then, the sections were treated with biotinylated anti-rabbit and anti-mouse secondary antibodies (Nichirei Biosciences Inc., Tokyo, Japan). Peroxidase activity was visualized with diaminobenzidine (DAB), using the DAB Substrate Kit (Nichirei Biosciences Inc.). The sections were observed and imaged using a Leica DMRBE microscope (Leica, Wetzlar, Germany) and a digital camera DP73 (Olympus, Tokyo, Japan). To evaluate the granulation tissue on the skin excision site, granulation tissue on peripheral wound and wound bed was observed (Fig. 2). These images were analyzed in Image J.

### 2.10 Statistical analysis

All results are expressed as mean ± standard error. A normality test (Shapiro–Wilk test) was conducted for all the data. Two-way ANOVA with post hoc test was used to analyze normally distributed data, and the Tukey–Kramer test was used to compare the two experimental groups. P < 0.05 was considered statistically significant.

## 3 Results

### 3.1 Fabrication of artificial amnion membrane

**Fig. 3:**
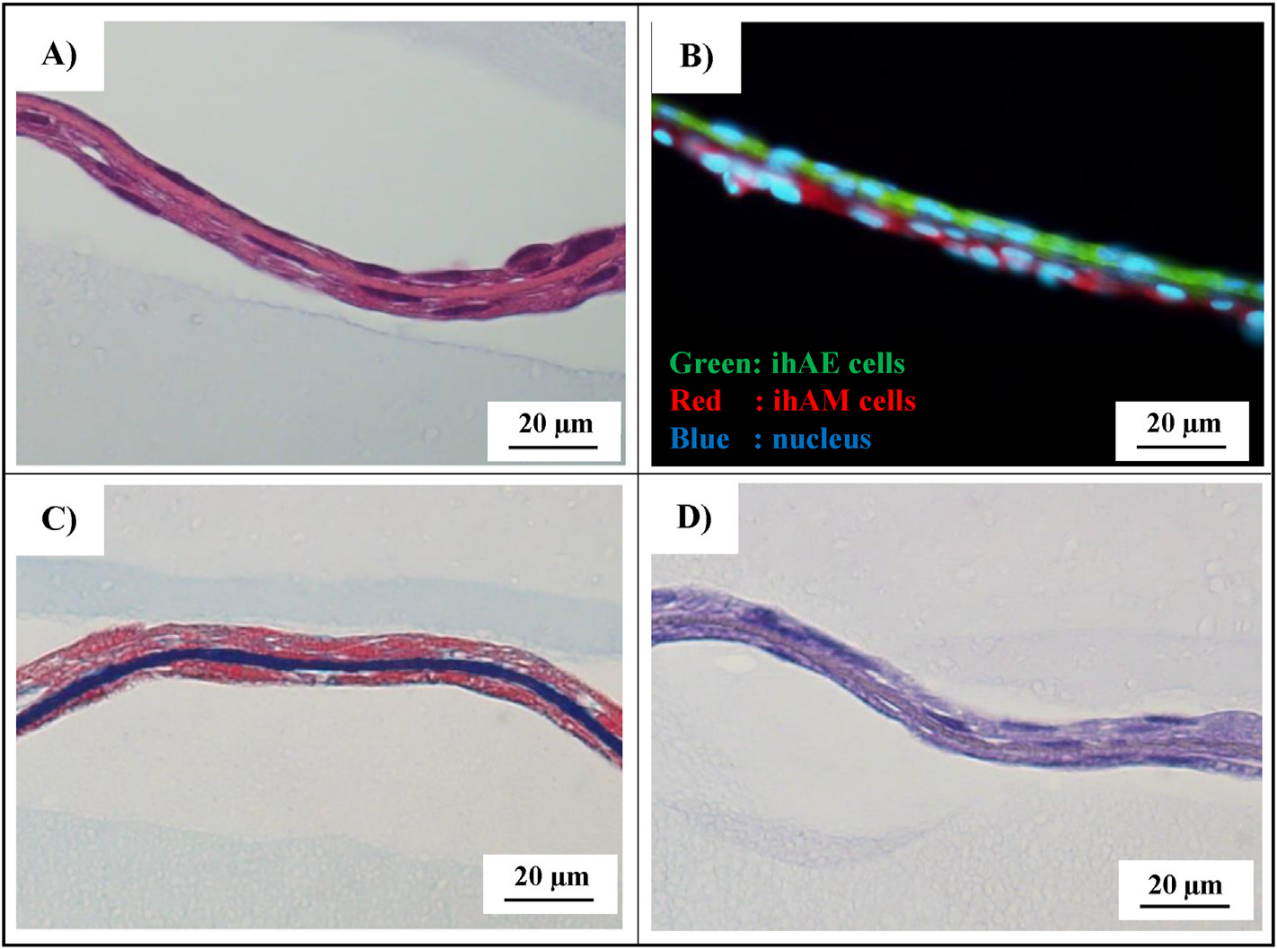
Cross-sectional view of artificial-AM using histological and immunofluorescence evaluation. (A) HE staining. (B) Fluorescence staining. (C) Azan staining. (D) Orcein staining. Scale bars, 20 μm.

The ihAE cells were attached to one side of the vitrigel, and the ihAM cells to the opposite side. Cross-sections of artificial AM showed that ihAE and ihAM adhered separately through the vitrigel (Fig. 3A and B). Azan staining revealed that the collagen fibers in vitrigel were highly dense (Fig. 3C). Orcein staining showed that the vitrigel did not contain elastic fibers (Fig. 3D).

### 3.2 Protein production capacity of artificial amnion membrane

**Fig. 4:**
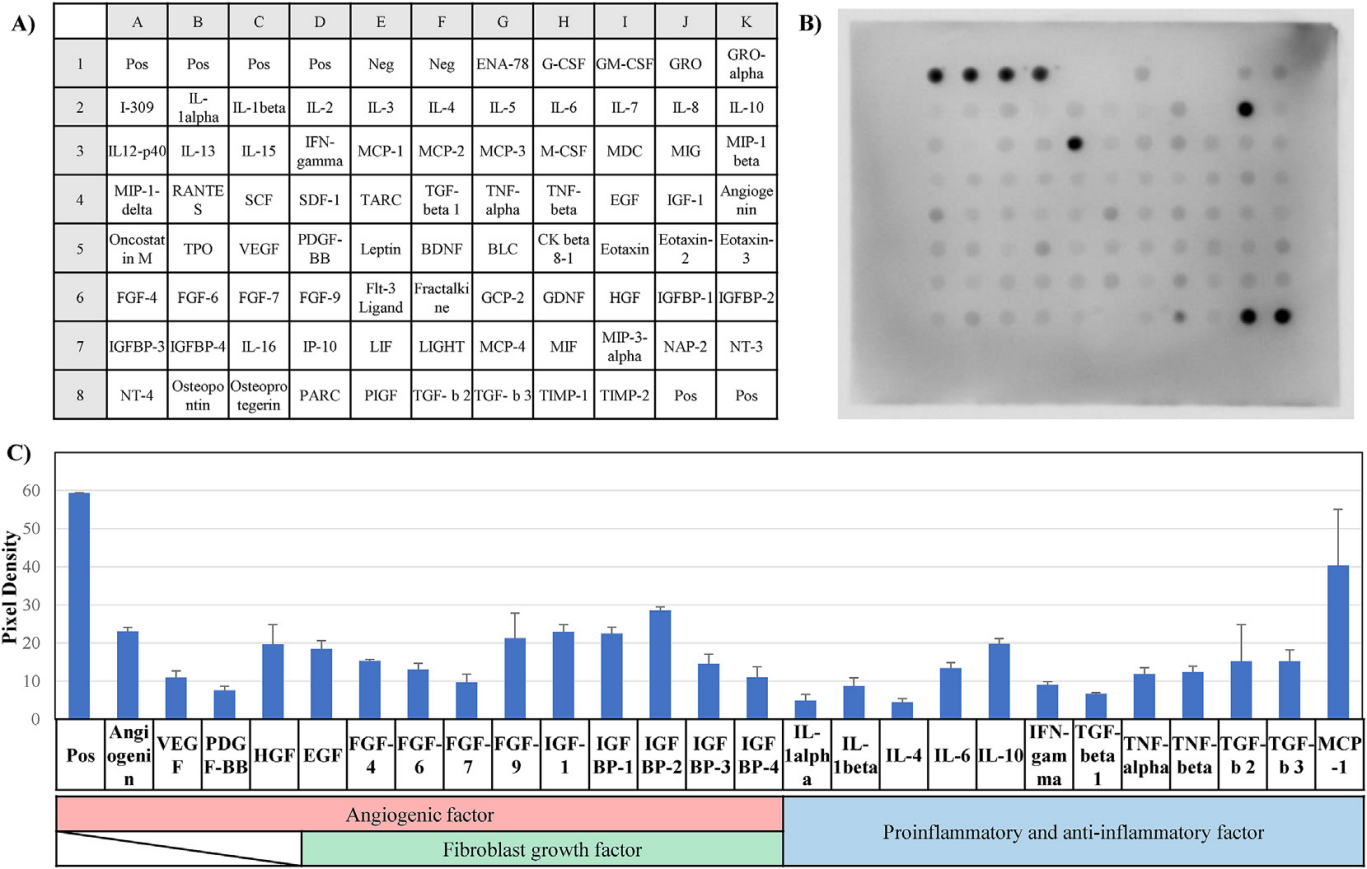
Protein array of the artificial-AM. (A) Measured protein list. (B) The protein array result of artificial-AM. (C) The expression levels of angiogenic factor, growth factor, and pro-inflammatory and anti-inflammatory factors. Data are expressed as means ± SE. (n = 3).

We analyzed 80 protein types extracted from the artificial AM (Fig. 4A and B). The proteins associated with angiogenic factor (angiogenin, VEGF, PDGF-BB, and HGF), fibroblast growth factor (EGF, FGF, IGF-1, and IGFBP), and proinflammatory and anti-inflammatory factors (IL-6, IL-10, TNFα, TGF-β, and MPC-1) were confirmed in the artificial AM (Fig. 4C).

### 3.3 *In vitro* evaluation of artificial amnion membrane

**Fig. 5:**
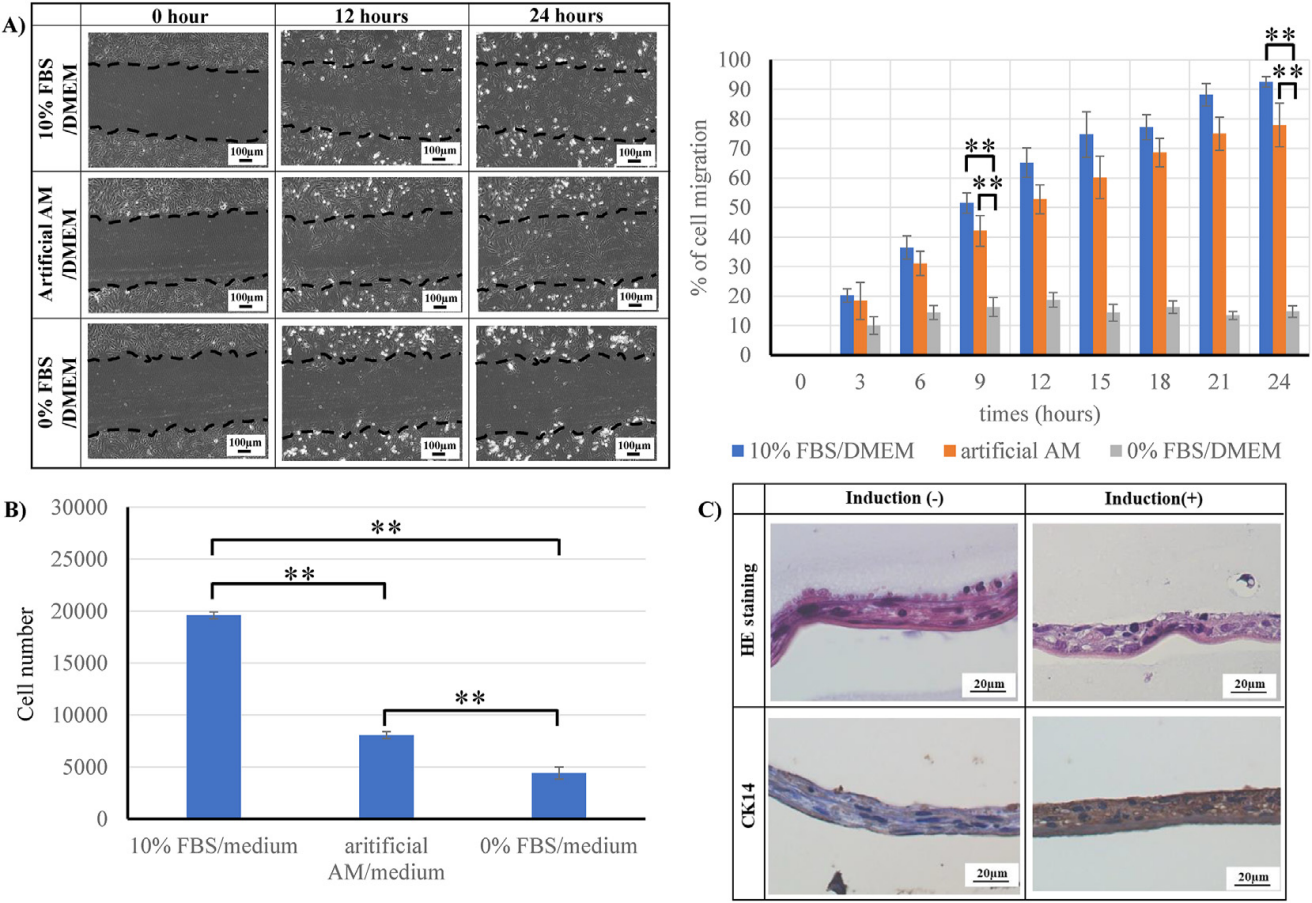
The functional evaluation of artificial-AM culture medium *in vitro*. (A) Time-lapse imaging of migration assay by HUVEC and analysis result. (B) Cell proliferation assay using HDFa cells. (C) Epidermal differentiation capacity evaluation. Data are expressed as means ± SE. (n = 3, ∗∗P < 0.01). Scale bars: 100 μm in (A, left panel) and 20 μm in (C).

The migration capacity of HUVECs was evaluated using a scratch assay. Migration was almost absent under the 0% FBS/DMEM condition. However, under 10% FBS/DMEM or artificial-AM culture conditions, the migration of HUVECs was observed (Fig. 5A). In addition, the proliferation of HDFa was evaluated in each medium. After 72 h of culture, HDFa could not proliferate in the 0% FBS/DMEM condition but proliferated in the artificial-AM culture medium (Fig. 5B). The epidermal differentiation capacity of ihAE cells on vitrigel was evaluated by the air-liquid interface system. Keratinocyte 14 was expressed in ihAE cells after the addition of induction factor of differentiation and air-liquid interface culture (Fig. 5C).

### 3.4 The transplantation of artificial amnion membrane to the third-degree burn injury diabetic mouse model

**Fig. 6:**
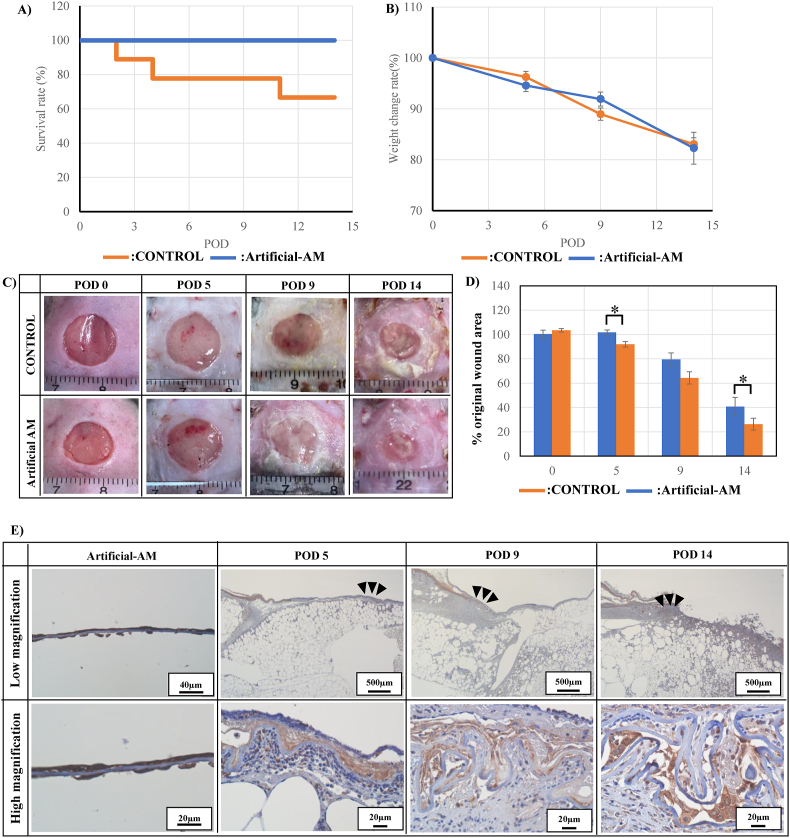
Transplantation of artificial-AM for the third-degree burn injury in diabetic mouse model. The survival rate (A) and weight change rate (B) of artificial-AM and control groups. Time-lapse images of wound closure rate (C) and analysis result (D) of each group over time. The artificial-AM engraftment was confirmed by CD59 staining (E). The high magnification image shows enlargement of the black arrows area in the low magnification image. Data are expressed as means ± SE. (n = 6, ∗P < 0.05). Scale bars: 20, 40, and 500 μm in (E).

The wound area in the artificial-AM group was covered by the artificial AM, whereas the control group received no such treatment. The mice died on POD 2, 4, and 11 in the control group, and the final survival rate of the control group was 66% on POD 14 (Fig. 6A). In contrast, all mice were alive in the artificial-AM group on POD 14. However, weight loss was observed in both groups at POD 5, 9, and 14 (Fig. 6B).

The wound site closure was observed on POD 5, 9, and 14. The artificial-AM group exhibited faster wound closure than the control group did (Fig. 6C and D). CD59 expression in the artificial-AM group was evaluated to confirm the engraftment of the transplanted artificial-AM in mice. The CD59 positive cells was confirmed in the artificial-AM group on POD 5, 9, and 14 (Fig. 6E).

### 3.5 Granulation tissue evaluation

**Fig. 7:**
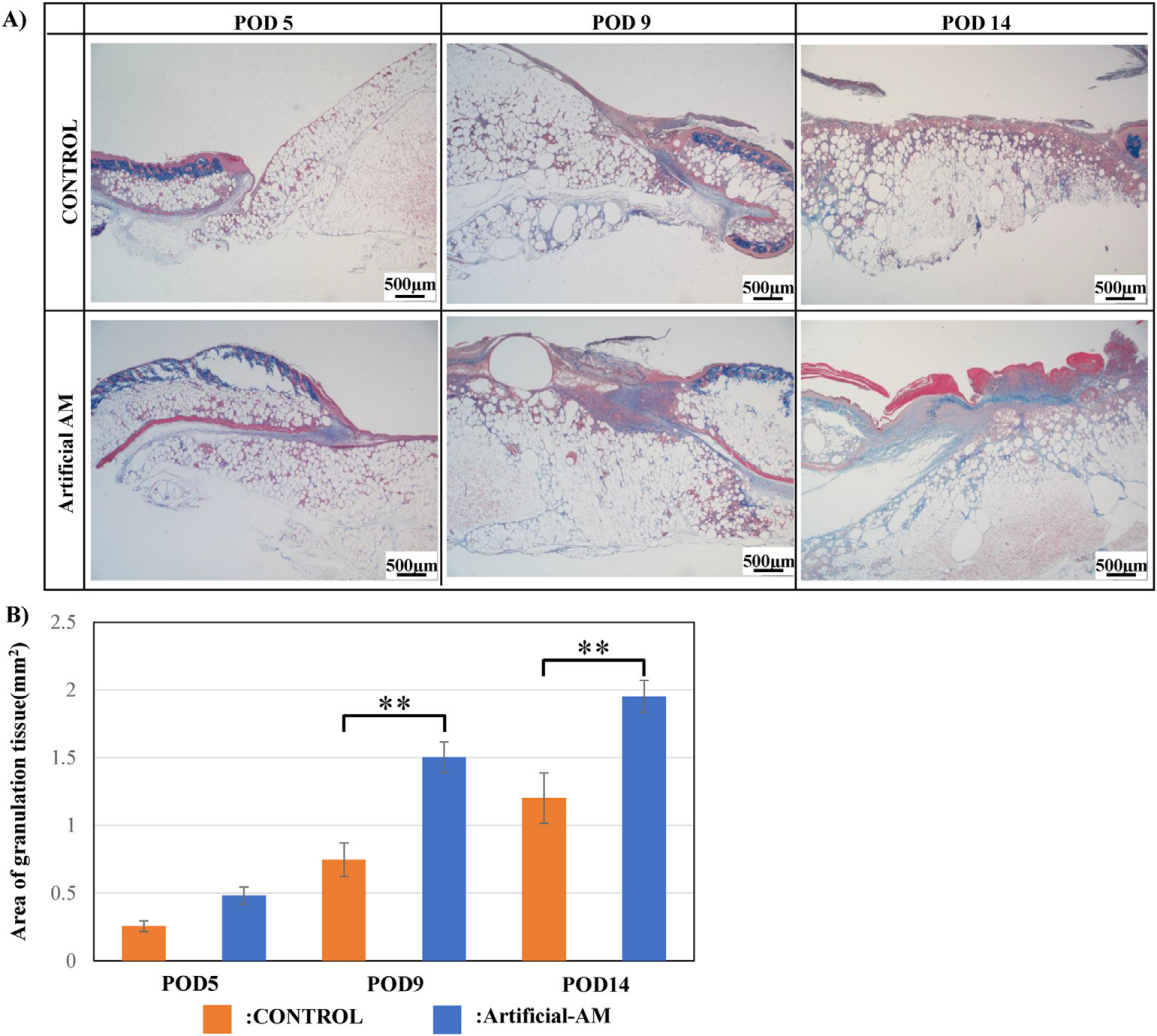
Evaluation of the granulation tissue area in the wound site. (A) Microscopic images of each group using azan staining. Scale bars, 500 μm. (B) The granulation tissue area of the artificial-AM and control groups Data are expressed as means ± SE. (n = 6, ∗∗P < 0.01).

To confirm wound healing in mice in the artificial-AM group, the area of the granulation tissue layer was visualized using azan staining (Fig. 7A). The granulation tissue was formed in both artificial-AM and control group over time (POD 5, 9, and 14). However, the region of granulation tissue in the artificial-AM group was significantly thicker and larger than that in the control group (Fig. 7B).

### 3.6 Wound healing effect of artificial AM on the third-degree burn injury diabetic mice model

**Fig. 8:**
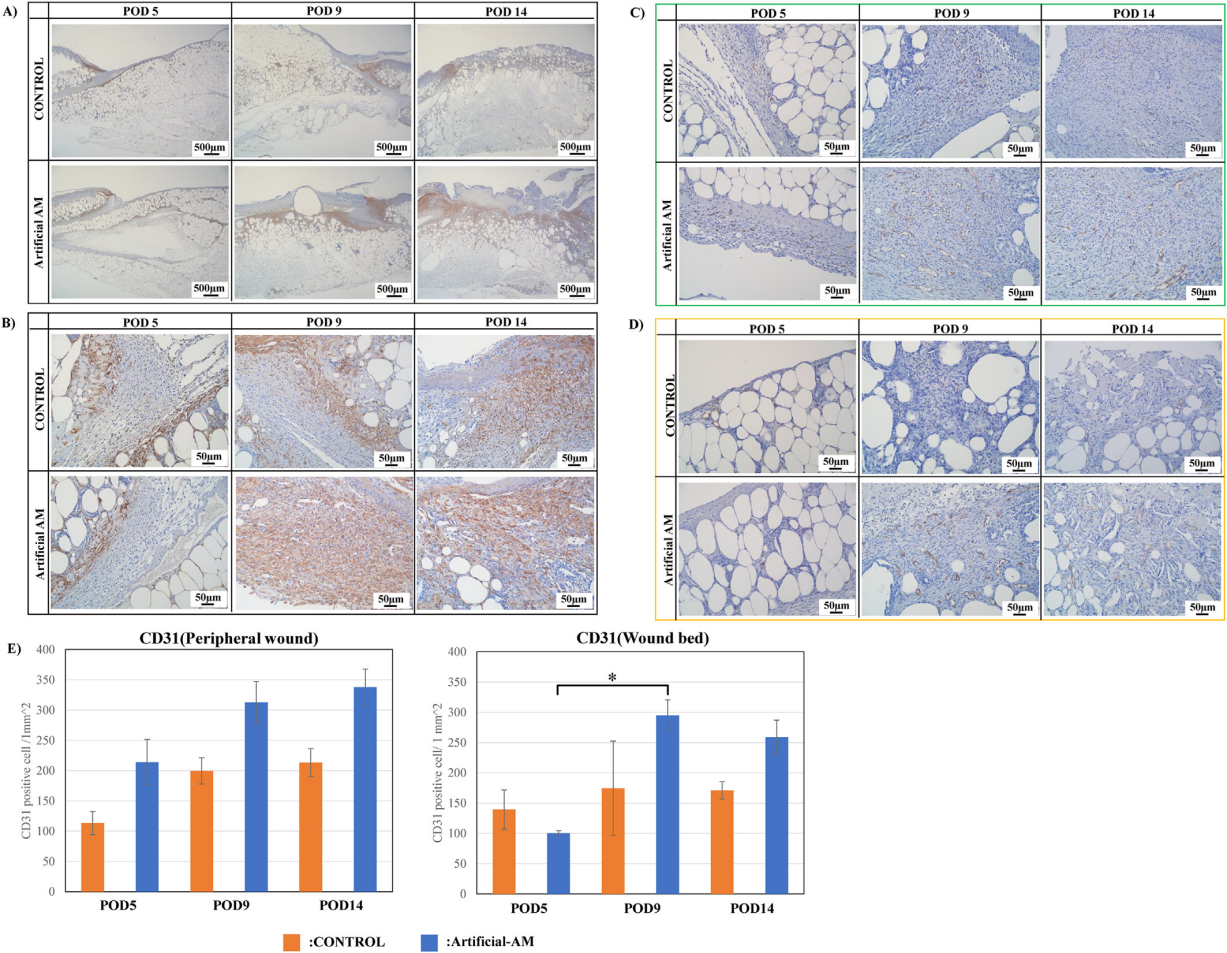
Immunostaining of α-SMA and CD31 expressions in granulation tissue. The α-SMA expression staining in granulation tissue at low (A) and high (B) magnification. The CD31 expression staining in granulation tissue was observed at peripheral wound (C) and wound bed (D). CD31 positive cells were measured and analyzed (E). Data are expressed as means ± SE. (n = 3, ∗P < 0.05). Scale bars: 500 μm in (A) and 50 μm in (B)–(C).

α-SMA positive cells in the granulation tissue were higher in the artificial-AM group than in the control group (Fig. 8A and B). To evaluate angiogenesis in granulation tissue, the presence of CD31 positive cells in the peripheral wound and wound bed was examined. CD31 positive cells in granulation tissue in peripheral wound of the artificial-AM group were higher than those of the control group from POD 5 to 14 (Fig. 8C and E). In granulation tissue of wound bed in the artificial-AM group, CD31 positive cells were increased only from POD 9 to 14 compared with that in the control group (Fig. 8D and E).

**Fig. 9:**
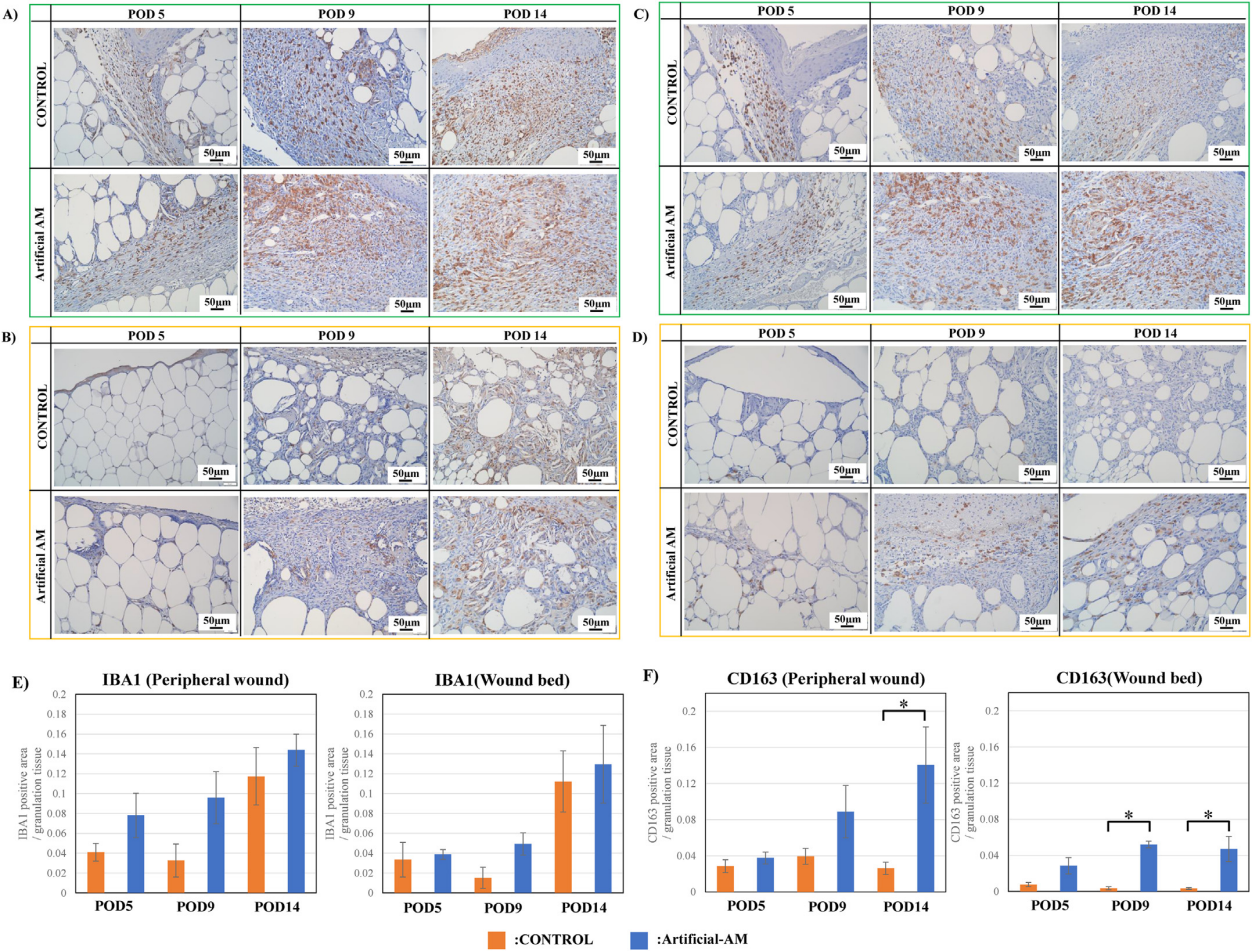
Immunostaining for IBA-1 and CD163 expressions in granulation tissue. IBA-1 expression staining in granulation tissue was observed at peripheral wound (A) and wound bed (B). CD163 expression staining in granulation tissue was observed at peripheral wound (C) and wound bed (D). IBA-1 expression area in peripheral wound and wound bed (E). The CD163 expression area in peripheral wound and wound bed (F). Data are expressed as means ± SE. (n = 3, ∗P < .05). Scale bars, 50 μm.

Next, the expression of IBA1 (M1 and M2 macrophages) and CD163 (M2 macrophage) was evaluated in granulation tissue. IBA1 positive cells in granulation tissue of peripheral wound were higher in the artificial-AM group than in the control group on POD 5 and 9 (Fig. 9A and E). Moreover, artificial-AM and control groups had higher IBA1 positive cells in peripheral wounds on POD 14 than on POD 9. Additionally, compared with the control group, artificial-AM group had increased IBA1 positive cells in granulation tissue of wound bed on POD 9 (Fig. 9B and E), and IBA1 positive cells in wound bed of artificial-AM and control groups had higher IBA1 positive cells in wound bed on POD 14 than on POD 9. In contrast, CD163 positive cells in granulation tissue of peripheral wound were significantly higher in artificial-AM group than in the control group on POD 9 and 14 (Fig. 9C and F). Moreover, in the control group, CD163 positive cells in granulation tissue of peripheral wound did not increase during POD 5 to 14. Although CD163 positive cells in granulation tissue of wound bed of artificial-AM group was observed on POD 5, they significantly increased only on POD 9 and 14 (Fig. 9D and F).

## 4 Discussion

The wound healing capacity of diabetes patients with third-degree burn injury is significantly impaired. Because their self-healing capacity is reduced, it is necessary to develop new dressing materials that promote wound healing for patients with diabetes. In this study, we designed an artificial AM using human AM-derived epithelial and mesenchymal cells and evaluated its wound healing-promoting effects. We then introduced growth factors produced from living amnion-derived cells into this artificial AM and evaluated its wound healing-promoting effects after implantation in a diabetic mouse model with third-degree burn. Amnion-derived epithelial cells prevent infections in injured wounds and produce several humoral factors. Amnion-derived mesenchymal cells can also produce anti-inflammation-related humoral factors [15,28,29]. Additionally, we used vitrigel as an extracellular matrix for attaching the epithelial and mesenchymal cells to the apical and basal surfaces to simulate an amnion membrane. The vitrigel is a gel composed of high-density collagen fibers produced by rehydration after the vitrification of collagen gel [23,24]. Therefore, artificial AM has higher mechanical strength than freeze-dried real amnion membrane. Moreover, we found that the artificial AM covering the third-degree burn wound site in diabetic mice was retained even on POD 14. Therefore, we inferred that there was a continuous supply of humoral factors from amnion membrane-derived cells in the artificial AM.

Next, we determined the types of humoral factors produced by the artificial AM. We found that angiogenic factors (angiogenin, VEGF, PDGF-BB, and HGF), fibroblast growth factors (EGF, FGF, IGF-1, and IGFBP), and proinflammatory and anti-inflammatory factors (IL-6, IL-10, TNFα, TGF-β, and MPC-1) were secreted from the artificial AM. To clarify the functional role of these humoral factors, we evaluated endothelial cell migration assays and fibroblast cell proliferation assays using culture supernatants of artificial AMs. We found that the culture supernatant promoted endothelial cell migration. Our results are consistent with the findings of Kim et al. who reported that AM-derived mesenchymal cells promote angiogenesis of vascular endothelial cells [29,30]. Previous studies have reported that AM-derived mesenchymal cells are capable of producing angiogenesis-related factors such as VEGF-A, Ang-1, HGF, and FGF-2 [30]. The present study also found that the artificial AM produced angiogenesis-related factors. Subsequently, we evaluated the cell proliferative ability of fibroblasts upon addition of culture supernatant and found that that after fibroblasts migrated and proliferated at the wound site, repair process was started and granulation tissue was formed via fibroblast extracellular matrix synthesis. Our findings demonstrated *in vitro* angiogenesis and fibroblast proliferation in the artificial AM.

To demonstrate the wound healing effect of artificial AM *in vivo*, we used a diabetic mouse model with third-degree burn. First, the ability of the artificial AM dressing to promote wound closure was evaluated. Wound closure was more accelerated in the artificial-AM group than in the control group, but complete wound closure could not be achieved by POD 14 in either group. In general, during the wound healing process, re-epithelialization begins after the formation of granulation tissue at the wound site [31]. However, because wound healing mechanism is inhibited in diabetic mice, re-epithelialization in diabetic mice is prolonged compared with that in normal mice [32]. In our study, α-SMA-positive cells (myofibroblasts) were identified after POD 9 in granulation tissue of the artificial-AM group. It has been reported that TGF-β produced from mesenchymal cells promotes the differentiation of fibroblast to myofibroblast and wound closure [33]. Thus, in the artificial-AM group, the differentiation of fibroblasts to myofibroblasts must have promoted wound contraction and closure, leading to early closure compared with the control group.

Next, the protein expressions of CD31, IBA1, and CD163 were evaluated to identify the cell types that migrate to the granulation tissue. To evaluate the differences in granulation tissue formation in different wound sites, we divided the wound site into peripheral wound (boundary between wound site and residual epidermis) and wound bed (epidermal defect site). CD31 immunostaining revealed that artificial-AM dressings promoted angiogenesis in the peripheral wound area and wound bed. Angiogenesis is important for the supply of oxygen and nutrients and the migration of immunocompetent cells to granulation tissue [34]. Because blood flow is impaired in patients with diabetes, the supply of oxygen and nutrients to wound site is hindered. Therefore, wound healing process in diabetes patients is often prone to infections and several other complex pathophysiology-related health issues.

In this study, we found that angiogenesis-related factors produced by artificial AMs promote the migration of vascular endothelial cells and angiogenesis. Subsequently, oxygen and nutrients are supplied to the wound site, resulting in the formation of thick granulation tissue. Macrophage dynamics analysis in granulation tissue revealed that the percentage of M2 macrophages in peripheral wound granulation tissue increased over time in the artificial-AM group. These results indicated that inflammation was promoted immediately after wound healing and rapidly shifted to the anti-inflammatory phase. Since artificial AMs produce both inflammatory cytokines (IL-1, IL-6, IFN-γ, and TNF-α) and anti-inflammatory cytokines (IL-10 and TGF-β), the balance of these cytokines may have contributed to the seamless progression of wound healing. Meanwhile, on POD 14, M2 macrophages as well as a higher number of M1 macrophages were observed in the wound bed, indicating a prolonged inflammatory phase in the wound bed. Recently, it has been reported that senescent cells in subcutaneous fat directly under the formed granulation tissue delay the wound healing process and prolong the inflammatory phase in diabetic mouse-wound model [35]. In this study, we hypothesize that the delayed wound healing process in the wound bed may have been caused by senescent cells in subcutaneous fat.

In summary, we were able to demonstrate the wound-healing effect of artificial AM on the third-degree burn injury diabetic mice model. However, it was difficult to use the artificial AM for long-term observation. After inducing third-degree burn injury, the body weight of the control and artificial AM groups decreased over time, and the survival rate of the control group decreased by POD 14. Iswara et al. reported that the body weight of non-diabetic mouse model does not change after wound injury, whereas that of diabetic mouse model decreases after wound injury [36]. We hypothesize that this weight loss may be related to hyperglycemia and impaired blood flow in the third-degree burn diabetic mouse model. Although changes in blood and body fluids (pH, specific gravity, blood gases, etc.) were not confirmed in this study, it may be necessary to consider the effect of a wound covering approximately 20% of the body surface on body fluids, since the artificial-AM group mice survived. The ihAE cells could be induced to differentiate into epidermal-like structures by air-liquid interface culture on ad-MED Vitrigel for different time points. In the future, if ihAE cells are induced to differentiate into epidermal-like structures *in vivo* by long-term dressing on artificial AMs, it is expected to be applied not only as a dressing material but also as artificial skin.

## 5 Conclusions

A reconstructed amniotic membrane structure was constructed in this study by seeding amnion-derived epithelial cells on one side of a vitrigel surface and mesenchymal stem cells on the opposite side. Our preliminary findings confirmed that the developed artificial AM produced multiple growth factors related to wound healing and showed functional effects *in vitro*. The artificial amnion exhibited the capacity to produce proteins and promote angiogenesis and cell proliferation. In addition, dressing of artificial AM in a third-degree burn diabetic mouse model revealed that it suppressed inflammation and promoted wound healing by enhancing anti-inflammation. The results demonstrated that amniotic membrane-based dressing material is a promising alternative to conventional wound dressings, as it can accelerate third-degree burn injury healing in preclinical and clinical setups. In the future, it is expected to be useful as a dressing material to promote wound healing of refractory and intractable ulcers.

## Declaration of competing interest

The authors declare that they have no conflict of interest.

## References

[1] Aldekhayel S., Khubrani A.M., Alshaalan K.S., Barajaa M., Al-Meshal O. (2021; Jun 15). Outcomes and complications of diabetic burn injuries: a single center experience. Int J Burns Trauma.

[2] Falanga V. (2005). Wound healing and its impairment in the diabetic foot. Lancet.

[3] Lau H.C., Kim A. (2016). Pharmaceutical perspectives of impaired wound healing in diabetic foot ulcer. J Pharm Investig.

[4] Faglia E., Favales F., Morabito A. (2001). New ulceration, new major amputation, and survival rates in diabetic subjects hospitalized for foot ulceration from 1990 to 1993: a 6.5-year follow-up. Diabetes Care.

[5] Ramsey S.D., Newton K., Blough D., McCulloch D.K., Sandhu N., Reiber G.E. (1999). Incidence, outcomes, and cost of foot ulcers in patients with diabetes. Diabetes Care.

[6] Noor S., Zubair M., Ahmad J. (2015). Diabetic foot ulcer—a review on pathophysiology, classification and microbial etiology. Diabetes Metabol Syndr.

[7] Kido D., Mizutani K., Takeda K., Mikami R., Matsuura T., Iwasaki K. (2017; Dec 21). Impact of diabetes on gingival wound healing via oxidative stress. PLoS One.

[8] Aitcheson S.M., Frentiu F.D., Hurn S.E., Edwards K., Murray R.Z. (2021 Aug 13). Skin wound healing: normal macrophage function and macrophage dysfunction in diabetic wounds. Molecules.

[9] Guo J., Hu H., Gorecka J., Bai H., He H., Assi R. (2018 Dec 1). Adipose-derived mesenchymal stem cells accelerate diabetic wound healing in a similar fashion as bone marrow-derived cells. Am J Physiol Cell Physiol.

[10] Assi R., Foster T.R., He H., Stamati K., Bai H., Huang Y. (2016 Apr). Delivery of mesenchymal stem cells in biomimetic engineered scaffolds promotes healing of diabetic ulcers. Regen Med.

[11] Oesman I., Dhamar Hutami W. (2020). Gamma-treated placental amniotic membrane allograft as the adjuvant treatment of unresponsive diabetic ulcer of the foot. Int J Surg Case Rep.

[12] Tseng S.C., Li D.Q., Ma X. (1999 Jun). Suppression of transforming growth factor-beta isoforms, TGF-beta receptor type II, and myofibroblast differentiation in cultured human corneal and limbal fibroblasts by amniotic membrane matrix. J Cell Physiol.

[13] Hao Y., Ma D.H., Hwang D.G., Kim W.S., Zhang F. (2000 May). Identification of antiangiogenic and antiinflammatory proteins in human amniotic membrane. Cornea.

[14] Marvin K.W., Keelan J.A., Eykholt R.L., Sato T.A., Mitchell M.D. (2002 Sep). Expression of angiogenic and neurotrophic factors in the human amnion and choriodecidua. Am J Obstet Gynecol.

[15] Buhimschi I.A., Jabr M., Buhimschi C.S., Petkova A.P., Weiner C.P., Saed G.M. (2004 Nov). The novel antimicrobial peptide beta3-defensin is produced by the amnion: a possible role of the fetal membranes in innate immunity of the amniotic cavity. Am J Obstet Gynecol.

[16] Koike N., Sugimoto J., Okabe M., Arai K., Nogami M., Okudera H. (2022 Jan 29). Distribution of amniotic stem cells in human term amnion membrane. Microscopy.

[17] Fenelon M., Maurel D.B., Siadous R., Gremare A., Delmond S., Durand M. (2019 Nov). Comparison of the impact of preservation methods on amniotic membrane properties for tissue engineering applications. Mater Sci Eng C.

[18] Perepelkin N.M., Hayward K., Mokoena T., Bentley M.J., Ross-Rodriguez L.U., Marquez-Curtis L. (2016 Mar). Cryopreserved amniotic membrane as transplant allograft: viability and post-transplant outcome. Cell Tissue Bank.

[19] Okabe M., Kitagawa K., Yoshida T., Suzuki T., Waki H., Koike C. (2014 Mar). Hyperdry human amniotic membrane is useful material for tissue engineering: physical, morphological properties, and safety as the new biological mater. J Biomed Mater Res.

[20] Zhou K., Koike C., Yoshida T., Okabe M., Fathy M., Kyo S. (2013 Feb). Establishment and characterization of immortalized human amniotic epithelial cells. Cell Reprogr.

[21] Teng Z., Yoshida T., Okabe M., Toda A., Higuchi O., Nogami M. (2013). Establishment of immortalized human amniotic mesenchymal stem cells. Cell Transplant.

[22] Li J., Koike-Soko C., Sugimoto J., Yoshida T., Okabe M., Nikaido T. (2015). Human amnion-derived stem cells have immunosuppressive properties on NK cells and monocytes. Cell Transplant.

[23] Moriyama M., Sahara S., Zaiki K., Ueno A., Nakaoji K., Hamada K. (2019 Dec 4). Adipose-derived stromal/stem cells improve epidermal homeostasis. Sci Rep.

[24] Yamaguchi H., Takezawa T. (2018 Nov). Fabrication of a corneal model composed of corneal epithelial and endothelial cells via a collagen vitrigel membrane functioned as an acellular stroma and its application to the corneal permeability test of chemicals. Drug Metab Dispos.

[25] Griffoni C., Neidhart B., Yang K., Groeber-Becker F., Maniura-Weber K., Dandekar T. (2021 Mar 29). In vitro skin culture media influence the viability and inflammatory response of primary macrophages. Sci Rep.

[26] Suarez-Arnedo A., Torres Figueroa F., Clavijo C., Arbeláez P., Cruz J.C., Muñoz-Camargo C. (2020 Jul 28). An image J plugin for the high throughput image analysis of in vitro scratch wound healing assays. PLoS One.

[27] Oba J., Okabe M., Yoshida T., Soko C., Fathy M., Amano K. (2020 Jul 27). Hyperdry human amniotic membrane application as a wound dressing for a full-thickness skin excision after a third-degree burn injury. Burns Trauma.

[28] Zheng Y., Zheng S., Fan X., Li L., Xiao Y., Luo P. (2018 Aug 19). Amniotic epithelial cells accelerate diabetic wound healing by modulating inflammation and promoting neovascularization. Stem Cell Int.

[29] Kim S.W., Zhang H.Z., Guo L., Kim J.M., Kim M.H. (2012). Amniotic mesenchymal stem cells enhance wound healing in diabetic NOD/SCID mice through high angiogenic and engraftment capabilities. PLoS One.

[30] Komaki M., Numata Y., Morioka C., Honda I., Tooi M., Yokoyama N. (2017 Oct 3). Exosomes of human placenta-derived mesenchymal stem cells stimulate angiogenesis. Stem Cell Res Ther.

[31] Ghiulai R., Roşca O.J., Antal D.S., Mioc M., Mioc A., Racoviceanu R. (2020 Nov 26). Tetracyclic and pentacyclic triterpenes with high therapeutic efficiency in wound healing approaches. Molecules.

[32] Uckun F.M., Orhan C., Tuzcu M., Durmus A.S., Ozercan I.H., Volk M. (2022 Jun 2). RJX improves wound healing in diabetic rats. Front Endocrinol.

[33] Putra A., Alif I., Hamra N., Santosa O., Kustiyah A.R., Muhar A.M. (2020 Dec 11). MSC-released TGF-β regulate α-SMA expression of myofibroblast during wound healing. J Stem Cells Regen Med.

[34] Beyer S., Koch M., Lee Y.H., Jung F., Blocki A. (2018 Sep 25). An in vitro model of angiogenesis during wound healing provides insights into the complex role of cells and factors in the inflammatory and proliferat. Int J Mol Sci.

[35] Kita A., Saito Y., Miura N., Miyajima M., Yamamoto S., Sato T. (2022 Apr 5). Altered regulation of mesenchymal cell senescence in adipose tissue promotes pathological changes associated with diabetic wound healing. Commun Biol.

[36] Iswara A., Tanaka K., Ishijima T., Nakajima Y., Mukai K., Tanaka Y. (2022 Oct 14). Wound healing in db/db mice with type 2 diabetes using non-contact exposure with an argon non-thermal atmospheric pressure plasma jet device. PLoS One.

